# Indole-3-Butyric Acid Induces Ectopic Formation of Metaxylem in the Hypocotyl of *Arabidopsis thaliana* without Conversion into Indole-3-Acetic Acid and with a Positive Interaction with Ethylene

**DOI:** 10.3390/ijms18112474

**Published:** 2017-11-21

**Authors:** Laura Fattorini, Federica Della Rovere, Eleonora Andreini, Marilena Ronzan, Giuseppina Falasca, Maria Maddalena Altamura

**Affiliations:** Dipartimento di Biologia Ambientale, Sapienza Università di Roma, 00185 Rome, Italy; laura.fattorini@uniroma1.it (L.F.); federica.dellarovere@uniroma1.it (F.D.R.); andreini.1468177@studenti.uniroma1.it (E.A.); marilena.ronzan@uniroma1.it (M.R.); giuseppina.falasca@uniroma1.it (G.F.)

**Keywords:** adventitious root formation, ethylene, hypocotyl pericycle, hypocotyl vascular system, indole-3-acetic acid, indole-3-butyric acid, metaxylem, protoxylem, xylogenesis in planta

## Abstract

The role of the auxins indole-3-acetic acid (IAA) and indole-3-butyric acid (IBA) and of the auxin-interacting phytohormone ethylene, on the ectopic formation of primary xylem (xylogenesis in planta) is still little known. In particular, auxin/ethylene-target tissue(s), modality of the xylary process (trans-differentiation vs. de novo formation), and the kind of ectopic elements formed (metaxylem vs. protoxylem) are currently unknown. It is also unclear whether IBA may act on the process independently of conversion into IAA. To investigate these topics, histological analyses were carried out in the hypocotyls of Arabidopsis wild type seedlings and *ech2ibr10* and *ein3eil1* mutants, which are blocked in IBA-to-IAA conversion and ethylene signalling, respectively. The seedlings were grown under darkness with either IAA or IBA, combined or not with the ethylene precursor 1-aminocyclopropane-1-carboxylic acid. Adventitious root formation was also investigated because this process may compete with xylogenesis. Our results show that ectopic formation of protoxylem and metaxylem occurred as an indirect process starting from the pericycle periclinal derivatives of the hypocotyl basal part. IAA favoured protoxylem formation, whereas IBA induced ectopic metaxylem with ethylene cooperation through the EIN3EIL1 network. Ectopic metaxylem differentiation occurred independently of IBA-to-IAA conversion as mediated by ECH2 and IBR10, and in the place of IBA-induced adventitious root formation.

## 1. Introduction

Xylogenesis consists of the ectopic formation of xylary elements [1]. The ectopic elements exhibit different thickenings in the secondary cell walls, i.e., the protoxylem-like elements show helical/annular thickenings, and the metaxylem-like elements pitted or densely reticulate thickenings, exactly as occurs in the normal primary vascular development in planta ([2], and references therein). The process of ectopic xylary element formation occurs when differentiated cells change their state, either directly trans-differentiating into xylary cells or indirectly, becoming xylary cells only after a dedifferentiation followed by a new differentiation event. In different species, different tissues may be engaged into xylary element formation, e.g., phloem parenchyma, pith parenchyma, vascular parenchyma, tuber parenchyma, mesophyll, and leaf epidermis [3]. However, in *Arabidopsis thaliana*, also the stem endodermis and the hypocotyl pericycle may be committed towards xylogenesis [1]. Xylogenesis also occurs in in vitro culture systems, and in the model plants *Zinnia elegans* and Arabidopsis it has been stressed that the xylary elements in vitro closely resemble the hypocotyl xylem components [1,4]. In addition, genes in common between non-ectopic xylem formation in planta and xylogenesis in vitro have been found [5], and transcriptional switches for protoxylem and metaxylem have been demonstrated in an Arabidopsis xylem formation system in vitro [6]. Moreover, even if transcription factors controlling proto-/metaxylem identity have been reported for Arabidopsis in planta [7], the identification of the molecular factors that are involved in determining xylem identity still remains an open question. The endogenous auxin level seems important in controlling the initiation of xylary elements and their size [8]. In accordance, it has been demonstrated that an endogenous auxin accumulation precedes the xylogenesis, and occurs independently of the fact that the process is preceded by a cell dedifferentiation event or results from a direct cell trans-differentiation [1,9,10]. 

In line with the prominent role of auxin in the process, both in planta and in in vitro culture, the ectopic formation of xylary cells is enhanced by exogenous auxins, alone or combined with cytokinin [1,3,10,11,12]. In addition, auxin affects the xylary identity, with the inhibition of auxin synthesis resulting into ectopic protoxylem formation in the place of metaxylem formation [13]. 

The most studied natural auxins are the indole-3-acetic acid (IAA) and its precursor indole-3-butyric acid (IBA). IAA is the main inducer of xylogenesis, e.g., in lettuce pith explants [14], tobacco pith explants, and thin cell layers [15], whereas IBA’s role still needs investigation. IAA is also an important inducer of adventitious root (AR) formation. However, IBA is more active in inducing AR formation, acting mainly by conversion into IAA by a β-oxidation process occurring in the peroxisomes ([16], and references therein), and by inducing genes of IAA biosynthesis [17]. It is unknown whether, when applied at the same concentration, exogenous IBA is more effective than exogenous IAA in enhancing xylogenesis and in determining xylary cell fate, and, if this is the case, whether IBA acts per se or by conversion into IAA.

To know the role of each auxin in xylogenesis is not only important for the lack of basic information about the process, but also for the possible biotechnological applications, e.g., for biofuel and biomaterials production [18].

Also, ethylene, which is known to be strictly related to IAA synthesis/action/transport [16,19], is involved in the xylogenic process. The positive involvement of ethylene in xylogenesis was demonstrated in lettuce pith explants that were treated with low doses of ethylene, ethylene releasing compounds, and precursors of ethylene, e.g., 1-aminocyclopropane-1-carboxylic acid (ACC), in the presence of IAA +/− kinetin [20]. The stimulation of xylem formation by exogenous ACC or by treatments with ethylene or ethylene-releasing compounds was then well documented in the stems or cuttings of numerous gymnosperms and other angiosperms [21,22,23,24]. However, only in recent years, the strict dependence of xylary differentiation on ethylene biosynthesis, and the stimulatory effect of this hormone on xylogenesis have been demonstrated [25]. A preferred approach in the study of ethylene role on xylogenesis is to utilize the mutants that are altered in ethylene biosynthesis and/or signalling [25]. The same approach has been recently used to investigate the role of ethylene on AR formation in Arabidopsis seedlings [16]. When applied at 0.1 μM, in combination with IBA (10 μM), ACC enhances AR formation, whereas it reduces when applied alone, but this does not occur in the *ein3eil1* ethylene-insensitive mutant. Moreover, the AR-response of the *ech2ibr10* mutant, blocked in the conversion of IBA into IAA, demonstrates that an ACC-enhanced IBA-to-IAA conversion is needed for AR formation [16]. It is known that mutations in genes encoding the enzymes specific for IBA-to-IAA conversion confer IBA resistance without altering the IAA response [26]. Candidates include ENOYL-COA HYDRATASE 2 (ECH2) and INDOLE-3-BUTYRIC ACID RESPONSE 10 (IBR10), both enoyl-CoA hydratase enzymes involved in peroxisomal IBA β-oxidation, and with the mutation in both the coding genes showing synergistic effects [27]. In fact, the *ech2ibr10* mutant is highly resistant to low IBA concentrations in dark-grown hypocotyl elongation assay and light-grown root elongation assay [27].

No information is available about the use of *ein3eil1* and *ech2ibr10* mutants in the study of xylogenesis. However, based on the steady state levels of the endogenous IAA and IBA in dark-grown Arabidopsis seedlings, and the changes in their levels that is caused by ACC [16], it is possible that ethylene is involved in the xylogenesis through the activation of transcription factors ETHYLENE INSENSITIVE 3 (EIN3) and EIN3-LIKE 1 (EIL1), as in the majority of the ethylene responses [28]. Both the transcription factors activate an array of primary ethylene response genes in the nucleus [28], and by the use of the *ein3eil1* mutant it has been demonstrated that the mutation in both of the factors results into an almost complete insensitivity to endogenous and exogenous (ACC-derived) ethylene ([16], and references therein). Moreover, also the IAA released by IBA through the activities of ECH2 and IBR10, might be important for xylogenesis, even if a role of IBA per se might not be excluded, as suggested for other processes [29]. 

All together, the research was aimed to determine the role of IAA, of its natural precursor IBA, and, of ethylene, on xylogenesis in the hypocotyls of Arabidopsis seedlings grown under darkness. The response of the double mutants *ech2ibr10* and *ein3eil1*, blocked in IBA-to-IAA conversion and in ethylene signalling, respectively, was compared with that of the wild type under the same treatments to identify the tissue(s) target of xylogenesis in planta, the modality of the xylary process (trans-differentiation or dedifferentiation followed by de novo xylary cell formation), and the kind of ectopic elements that are formed. The evaluation of ARs and AR primordia (ARPs) was also carried out, because they might either positively affect the xylogenic response, being active sites of IAA synthesis/accumulation [1,30,31], or negatively affect it because AR formation occurs in competition with xylogenesis in numerous culture systems ([12], and references therein).

Our results show that the ectopic formation of protoxylem and metaxylem occurred only in the basal hypocotyl, and was an indirect process starting from pericycle periclinal derivatives. IAA favoured protoxylem formation, and protoxylem was formed to connect the ARPs with the parental vasculature. By contrast, IBA induced ectopic metaxylem, with the cooperation of ethylene through the EIN3EIL1 network, and as an alternative of AR formation. The role of IBA on ectopic metaxylem formation occurred through a pathway that was independent of the IBA-to-IAA conversion that was mediated by ECH2 and IBR10.

## 2. Results

### 2.1. The Hypocotyl Pericycle Shows Periclinal Divisions Leading to Ectopic Metaxylem Formation in the Presence of Indole-3-Butyric Acid (IBA)

**Figure 1:**
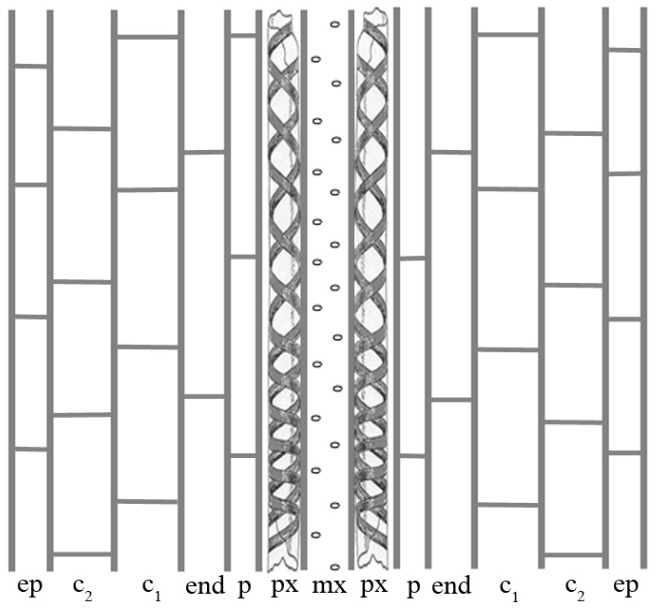
Scheme representing the primary xylem (protoxylem (px) and metaxylem (mx)) of the basal hypocotyl of 22 days after stratification (DAS) *Arabidopsis thaliana* dark-grown seedlings in longitudinal section. The tissues external to the xylem are indicated by the abbreviations: ep, epidermis; c_1_ and c_2_, cortical layers; end, endodermis; p, perycicle.

The primary vasculature of the hypocotyl in Arabidopsis intact seedlings is characterized by rows of xylem elements with exarch protoxylem and endarch metaxylem [32], and, as in the primary root, the vasculature is surrounded by a pericycle layer, as exemplified in Figure 1. The hypocotyl base is the region confining with the primary root, and is the site where endogenous auxin accumulates, and rare ARPs are formed there by anticlinal divisions of the pericycle followed by periclinal divisions leading to the root dome formation [30].

**Figure 2:**
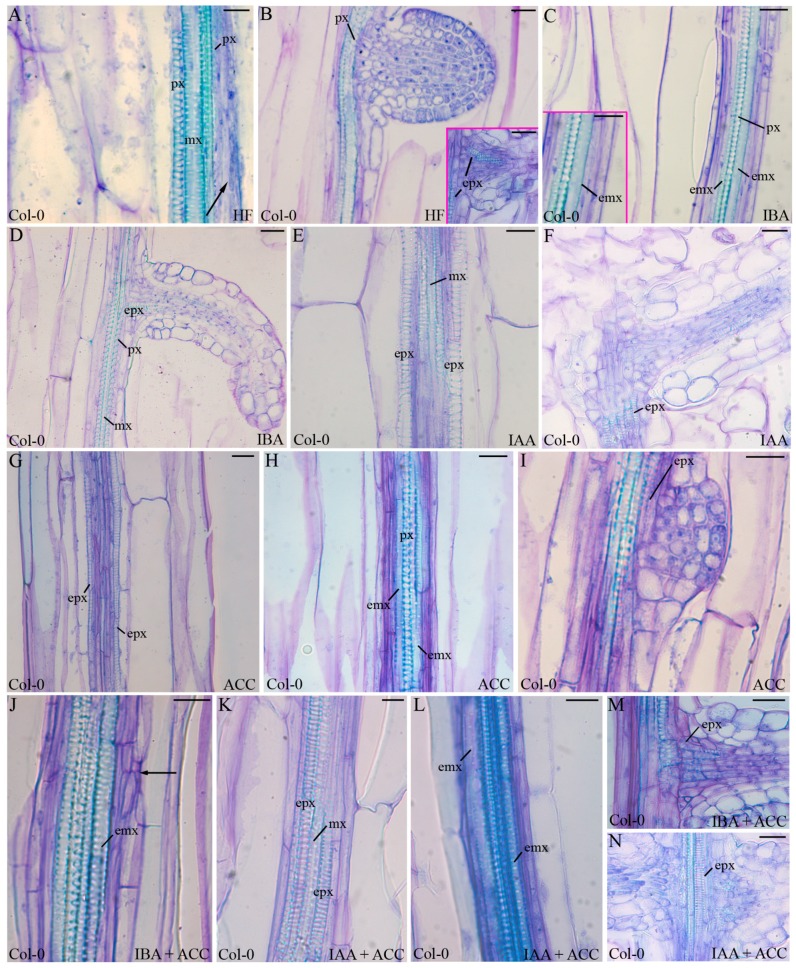
Xylogenesis and adventitious root (AR) formation in the pericycle proliferation at the base of the hypocotyl of 22 days after stratification (DAS) Col-0 (wild type) seedlings grown under darkness and different hormonal conditions. (**A**) periclinal proliferation in the pericycle (arrow), and (**C**,**E**,**G**,**H**,**J**,**K**,**L**, and Inset in **C**) ectopic xylary element formation in this proliferation. (**B**,**D**,**F**,**I**,**M**,**N**, and Inset in **B**) xylary connection between the AR primordium and the hypocotyl vasculature. (**A**,**B**, and Inset in **B**) hormone free (HF), (**C**,**D**, and Inset in **C**) indole-3-butyric acid (IBA) at 10 μM, (**E**,**F**) indole-3-acetic acid (IAA) at 10 μM, (**G**–**I**) 1-aminocyclopropane-1-carboxylic acid (ACC) at 0.1 μM, (**J**,**M**) IBA (10 μM) + ACC (0.1 μM), (**K**,**L**,**N**) IAA (10 μM) + ACC (0.1 μM). emx, ectopic metaxylem; epx, ectopic protoxylem; mx, metaxylem; px, protoxylem. Longitudinal sections stained with toluidine blue. Scale bars = 10 μm (**K**), 20 μm (**A**,**C**,**E**,**F**,**H**–**J**,**L**–**N**, Inset in **C**), 30 μm (**B**,**D**,**G**, Inset in **B**).

In seedlings grown under continuous darkness for 22 days after stratification (DAS) without exogenous hormones (hormone free (HF) treatment) the vascular patterning of the hypocotyl always showed exarch protoxylem, even if, at the hypocotyl base, the pericycle occasionally showed periclinal cell proliferation (Figure 2A, arrow). The vasculature below the ARPs always showed exarch protoxylem (Figure 2B), and the vascular connection between the ARP and the parental vasculature was formed by protoxylem-like elements (Figure 2B Inset).

**Figure 3:**
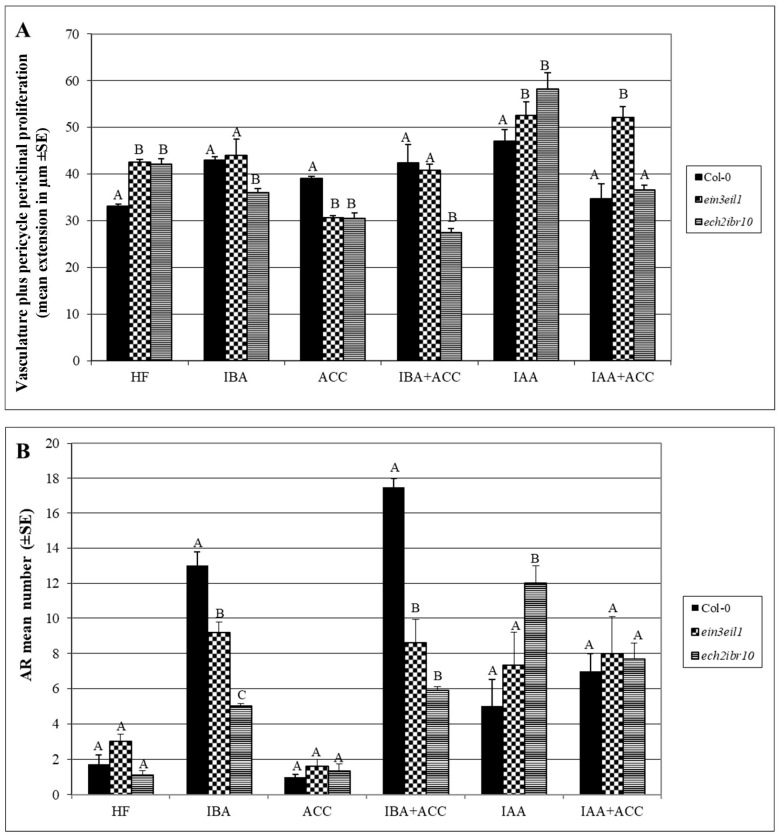
Radial extension (μm) of the vascular system, including the de novo formed cells by pericycle periclinal proliferation, at the middle of the 5-mm-basal portion of the hypocotyl (**A**), and number of adventitious roots (ARs), including AR primordia, (**B**) in the entire 5-mm-basal portion of the hypocotyl of 22 DAS seedlings of Col-0 (wild type), *ein3eil1* and *ech2ibr10* genotypes grown under darkness and different hormonal conditions (hormone free (HF), 10 μM indole-3-butyric acid (IBA), 0.1 μM 1-aminocyclopropane-1-carboxylic acid (ACC), 10 μM IBA + 0.1μM ACC (IBA + ACC), 10 μM indole-3-acetic acid (IAA), 10 μM IAA + 0.1 μM ACC (IAA + ACC)). Mean values (±SE) from sections observed under light microscopy. Different letters indicate significant differences between genotypes within the same treatment, at least at *p* < 0.05 level. Significant differences between treatments within the same genotype, and further statistical details, are described in the text. *n* = 30.

**Figure 4:**
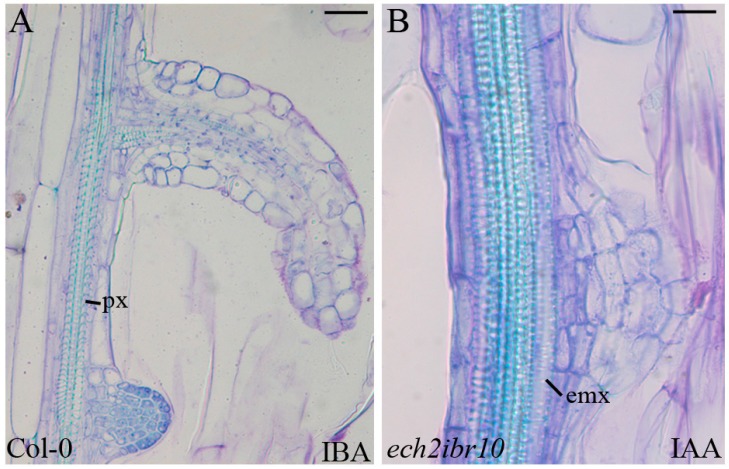
Vascular zone in the basal hypocotyl of 22-DAS dark-grown seedlings of *Arabidopsis thaliana*. (**A**) vascular region between confining adventitious root primordia (ARPs) with external protoxylem (px) (Col-0, wild type, at 10 μM IBA). (**A**) is a larger hypocotyl portion of Figure 2D. (**B**) ectopic metaxylem (emx) formation rarely occurring under ARPs (*ech2ibr10* mutant, 10 μM IAA treatment). Longitudinal sections stained with toluidine blue. Scale bars = 20 μm (**B**), 40 μm (**A**).

The treatment with IBA stimulated the pericycle to divide periclinally in comparison with HF, as shown by the significant increase (*p* < 0.05) in the radial extension of the vascular zone quantified in Figure 3A. The meristematic cells derived from these periclinal derivatives further developed into ectopic metaxylem elements (Figure 2C). The metaxylem elements in contact with the original protoxylem matured before the more external elements (Figure 2C and Inset). These latter elements remained immature, with a not yet lignified cell wall, but with pitted secondary thickenings. The IBA-treatment stimulated AR formation in comparison with HF and IAA (*p* < 0.0001 difference with both treatments, Figure 3B). The hypocotyl vasculature was regular (i.e., with exarch protoxylem) in the sites where the IBA-induced ARPs were formed, and the vascular connection of the ARP with the hypocotyl was always formed by protoxylem-like tracheary elements (Figure 2D). Moreover, also the hypocotyl between confining ARPs exhibited a regular vasculature with exarch protoxylem (Figure 4A, showing a larger part of Figure 2D). The treatment with IAA caused a highly significant (*p* < 0.0001) increase in pericycle periclinal proliferation in comparison with HF (Figure 3A), however only ectopic protoxylem was formed (Figure 2E) and the AR number did not increase significantly (Figure 3B). Also, in the case of IAA treatment, the vascular connection of the ARPs with the parental vasculature was formed by protoxylem-like cells (Figure 2F).

Collectively, results show that exogenous IBA stimulated AR formation, and much more than IAA (*p* < 0.0001 difference), but where ARPs were not formed, IBA caused ectopic metaxylem formation, whereas exogenous IAA stimulated protoxylem formation.

### 2.2. The Hypocotyl Pericycle Shows Periclinal Divisions Leading to Both Ectopic Metaxylem and Ectopic Protoxylem in the Presence of 1-Aminocyclopropane-1-Carboxylic Acid (ACC)

Being the ethylene precursor, ACC is frequently applied exogenously as an experimental treatment for investigating ethylene responses ([33], and references therein). In the present research, ACC was applied at the same concentration that was adopted for investigating AR formation in the hypocotyls of Arabidopsis seedlings grown under the same conditions [16]. In the basal portion of the hypocotyl, the ACC treatment caused a slight pericycle periclinal proliferation, which was not significantly different from that present under HF (Figure 3A), however, either ectopic protoxylem (Figure 2G) or ectopic metaxylem (Figure 2H) formation occurred in different seedlings. Only rare ARs were formed, as under HF (Figure 3B), and, interestingly, the vascular connection of the ARP was formed by only protoxylem-like cells (Figure 2I), as in the case of each auxin treatment.

When ACC was combined with IBA, the pericycle periclinal divisions continued to appear at the hypocotyl base, forming a proliferation zone that was not significantly different, in radial extension, to that observed under IBA alone (Figure 3A), and only ectopic metaxylem was formed, as in the case of IBA alone (Figure 2J,C, in comparison). However also anticlinal divisions frequently appeared in the pericycle derivatives (Figure 2J, arrow), and gave rise to a conspicuous AR formation, which was higher (*p* < 0.05) than under IBA alone (Figure 3B), in accordance with previous data on entire seedlings [16]. The combined presence of IAA and ACC caused again the pericycle periclinal proliferation, which was not significantly different from that observed under ACC alone (Figure 3A). Either ectopic protoxylem or ectopic metaxylem was formed, as observed for ACC alone (Figure 2K,L and G,H, in comparison).

The vascular connection of the ARPs was formed exclusively by protoxylem-like elements, both in the presence of IBA + ACC, and IAA + ACC (Figure 2M,N), and the hypocotyl vascular zone between confining ARPs always showed external protoxylem, as in the case of IBA alone (Figure 4A).

All together, results show that ectopic metaxylem is also caused by ethylene. The combined presence of ACC and exogenous IBA enhances this morphogenic event, however it also stimulates AR formation, which, instead, is always associated to protoxylem differentiation. 

### 2.3. The Xylogenic Response of the ein3eil1 Double Mutant Supports that Ethylene Signalling Is Necessary for Ectopic Metaxylem Formation 

**Figure 5:**
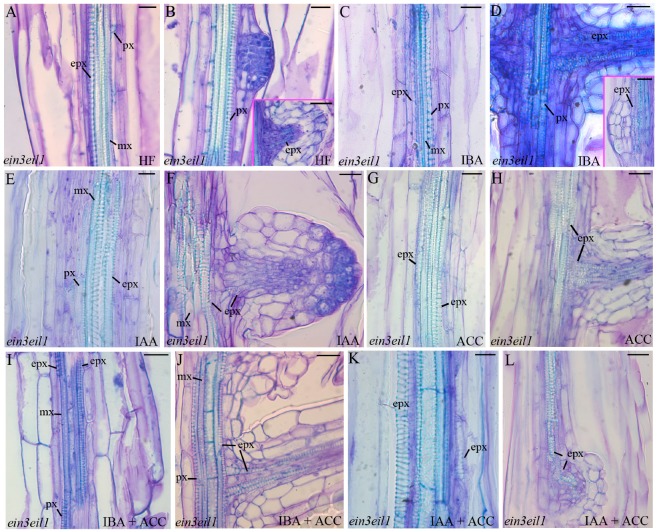
Xylogenesis and adventitious root (AR) formation in the pericycle proliferation at the base of the hypocotyl of 22 DAS *ein3eil1* seedlings grown under darkness and different hormonal conditions. (**A**,**C**,**E**,**G**,**I**,**K**) ectopic xylary element formation. (**B**,**D**,**F**,**H**,**J**,**L**, and Insets in **B**,**D**) xylary connection between the AR primordium and the hypocotyl vasculature. (**A**,**B**, and Inset in **B**) hormone free (HF), (**C**,**D**, and Inset in **D**) indole-3-butyric acid (IBA) at 10 μM, (**E**,**F**) indole-3-acetic acid (IAA) at 10 μM, (**G**,**H**) 1-aminocyclopropane-1-carboxylic acid (ACC) at 0.1 μM, (**I**,**J**) IBA (10 μM) + ACC (0.1 μM), (**K**,**L**) IAA (10 μM) + ACC (0.1 μM). emx, ectopic metaxylem; epx, ectopic protoxylem; mx, metaxylem; px, protoxylem. Longitudinal sections stained with toluidine blue. Scale bars = 20 μm (**A–D**,**F–J**,**L**, Insets in **B**,**D**), 40 μm (**E**,**K**).

In general accordance with the auxin-sensitivity of *ein3eil1* [34], both AR formation ([16], and Figure 3B) and xylogenesis (Figure 5) occurred. Regarding ARs/ARPs, their vascular connection with the parental vasculature again occurred by protoxylem-like elements independently of the treatment (Figure 5B,D,F,H,J,L). 

In the basal hypocotyl, when ACC alone was applied, the pericycle showed a periclinal proliferation that was significantly (*p* < 0.05) reduced in comparison with the wild type (Figure 3A). In this mutant, the application of IAA, and of IAA + ACC, without any significant difference between the two, enhanced (*p* < 0.05) the pericycle periclinal proliferation in comparison with HF (Figure 3A). Interestingly, independently of the treatment, ectopic metaxylem never appeared (Figure 5A,C,E,G,I,K). Even if unable to induce ectopic metaxylem, the mutant remained able to form ARs in the basal hypocotyl. The IBA and IBA + ACC treatments induced a number of ARs/ARPs significantly (*p* < 0.001) higher than HF, even if significantly lower than in the wild type under the same hormonal conditions (*p* < 0.05 and *p* < 0.0001, respectively) (Figure 3B). The presence of an AR response was in accordance with previous results in the entire hypocotyls of the same genotype under the same treatments [16], but interestingly, the AR-response was similar under both of the exogenous auxins (±ACC) (Figure 3B).

All together, the results about the ectopic metaxylem response of this mutant show that the ACC effect occurs through EIN3EIL1 network, and that the promotion by IBA of ectopic metaxylem formation requires this network. Moreover, results show that the inactivation of the EIN3EIL1 network also reduces the exogenous IBA-induced AR formation.

### 2.4. The Xylogenic Response of the ech2ibr10 Double Mutant Shows that the Conversion of IBA into Indole-3-Acetic Acid (IAA) Is Not Necessary to IBA-Induced Metaxylem Formation

Figure 3B shows that in the basal hypocotyl of the *ech2ibr10* mutant, blocked in IBA-to-IAA conversion, the formation of ARs/ARPs was similar to that of the wild type under HF or ACC treatments, whereas it became reduced with IBA treatments or enhanced with IAA. This shows the presence of an IAA-responsiveness for AR formation in this mutant, in accordance with its known IAA-sensitivity [26], and confirms the importance of an active IBA-to-IAA conversion for AR formation in Arabidopsis [16,17]. However, ACC significantly reduced (*p* < 0.05) the AR-response when combined with IAA in comparison with IAA alone (Figure 3B), suggesting a different action of ethylene on AR formation, depending on the exogenous auxin partner (IAA or IBA).

**Figure 6:**
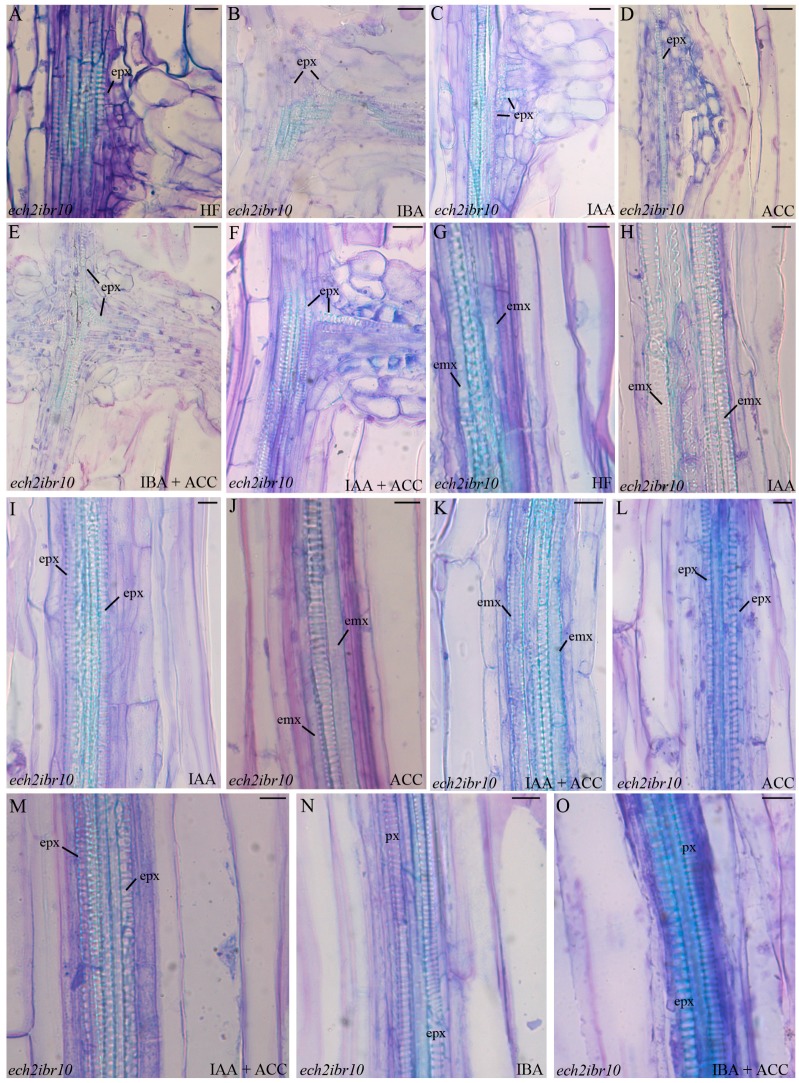
Xylogenesis and adventitious root (AR) formation in the pericycle proliferation at the base of the hypocotyl of 22 DAS *ech2ibr10* seedlings grown under darkness and different hormonal conditions. (**A**–**F**) Xylary connection between the AR primordium and the hypocotyl vasculature. (**G**–**O**) ectopic xylary element formation. (**A**) hormone free (HF), (**B**) indole-3-butyric acid (IBA) at 10 μM, (**C**) indole-3-acetic acid (IAA) at 10 μM, (**D**) 1-aminocyclopropane-1-carboxylic acid (ACC) at 0.1 μM, (**E**) IBA (10 μM) + ACC (0.1 μM), (**F**) IAA (10 μM) + ACC (0.1 μM), (**G**) HF, (**H**,**I**) IAA (10 μM), (**J**,**L**) ACC (0.1 μM), (**K**,**M**) IAA (10 μM) + ACC (0.1 μM), (**N**) IBA (10 μM), (**O**) IBA (10 μM) + ACC (0.1 μM). emx, ectopic metaxylem; epx, ectopic protoxylem; mx, metaxylem; px, protoxylem. Longitudinal sections stained with toluidine blue. Scale bars = 10 μm (**A**–**C**,**F**,**I**,**K**,**L**,**N**), 20 μm (**D**,**E**,**G**,**H**,**J**,**M**,**O**).

The vascular connection of the ARP with the parental organ usually occurred by de novo formation of protoxylem-like elements (Figure 6A–F). However, occasionally, metaxylem-like elements occurred under the ARP (Figure 4B).

The pericycle periclinally proliferated under all of the treatments, but highly under IAA alone (*p* < 0.0001 in comparison with the other treatments), and poorly under IBA + ACC (Figure 3A). Moreover, ACC alone reduced (*p* < 0.01) the proliferation in this mutant like in *ein3eil1* (Figure 3A). Interestingly, under HF conditions, ectopic metaxylem formation appeared in the basal periclinal proliferation of about 30% of the *ech2ibr10* hypocotyls (Figure 6G). Ectopic metaxylem also occurred at the hypocotyl base of a few (10% by average) IAA-alone treated seedlings (Figure 6H). By contrast, the majority of the mutant seedlings under this treatment showed ectopic protoxylem formation (Figure 6I), as in the wild type under the same treatment (Figure 2E), again in accordance with the mutant IAA-sensitivity [26]. *ech2ibr10* is also known to be ethylene-sensitive [16], and, in accordance, about the half of the seedlings treated with either ACC, or ACC combined with IAA, showed ectopic metaxylem formation (Figure 6J,K), whereas the other half showed ectopic protoxylem formation (Figure 6L,M).

Taken together, the results suggest that the endogenous IBA might cause ectopic metaxylem formation acting independently of its conversion into IAA, with ethylene positively contributing to enhance the process.

In comparison with the other genotypes, the treatments with exogenous IBA showed a significantly (*p* < 0.05 for IBA, and *p* < 0.0001 for IBA + ACC) reduced periclinal proliferation of the basal pericycle (Figure 3A), with no ectopic metaxylem formation, but a normal vasculature with exarch protoxylem (Figure 6N,O). In the same two treatments, even if poor, the AR formation was higher than under HF (*p* < 0.05 for IBA and *p* < 0.01 for IBA + ACC, Figure 3B), suggesting that independently of its conversion into IAA, IBA was able per se to induce at least in part AR formation, with this action inhibiting metaxylem formation as an alternative program.

## 3. Discussion

Our results show that IBA, up to now being considered as a natural precursor of IAA, is able to induce ectopic metaxylem formation, with the cooperation of ethylene, and that the ectopic metaxylem formation occurs in the place of AR formation. The role of IBA on ectopic metaxylem occurs through a pathway independent of the IBA-to-IAA conversion. 

### 3.1. IBA Is Determinant for the Equilibrium between Ectopic Metaxylem Development and Adventitious Root (AR) Formation in the Hypocotyl Pericycle

The pericycle at the base of the hypocotyl is the favoured site of the formation of one-two ARs in Arabidopsis seedling grown under HF conditions [11]. The AR response highly increases, appearing all along the hypocotyl, under the input of IBA (10 μM) plus kinetin (0.1 μM), but in both conditions (HF and IBA + Kin), anticlinal divisions must occur in the founding pericycle cells to generate the ARP [30]. However, under the IBA + Kin treatment, at the base of the hypocotyl (junction between this organ and the primary root), the pericycle cells sporadically divide according to periclinal division planes, generating layers of meristematic derivative cells further differentiating into ectopic metaxylem [1]. This means that the same pericycle cells may be primed toward either AR formation or xylogenesis by the IBA + Kin treatment, with this priming effect being associated with a different orientation (anticlinal and periclinal, respectively) of the cytoskeleton involved in cytokinesis [1]. Changes in cytoskeleton orientation also negatively affect lateral root formation, as in Arabidopsis *wooden leg* (*wol*) mutant. The seedlings of the *wol* mutant show roots and basal hypocotyls with all the vascular cells differentiated into xylem elements [35]. Microscopic observations of the *wol* primary root reveal that the pericycle cells adjacent to the protoxylem are able to divide in a periclinal way upon auxin treatment, but, in this case, they do not form lateral root primordia [36]. Present data add new information, because they show that IBA, applied alone to seedlings grown under the same cultural conditions previously used [1,31], is able to cause both AR formation and ectopic metaxylem formation. Moreover, ethylene cooperates with IBA in both events. The easiest interpretation of IBA action is that it is converted into IAA, and that IAA is responsible for both processes. IAA has in fact a well known inductive action on both AR formation and xylogenesis ([12], and references therein). The fact that IBA may have functions that are independent of its conversion into IAA has been the subject of a long debate [37]. However, present data of *ech2ibr10* mutant, blocked in the IBA-to-IAA conversion, support that IBA may contribute to AR formation also independently of conversion into IAA (Figure 3B), but, mainly, show a new, IAA-independent, role of IBA on ectopic metaxylem formation (Figure 6).

It is interesting to observe that even if the ectopic metaxylem formation is an indirect process, i.e., cell-proliferation-mediated, the periclinal division activity by the pericycle is necessary, but not sufficient, for its realization. In fact, the pericycle proliferates under all treatments and genotypes (Figure 3A), but mainly under IAA, i.e., in a treatment unable (wild type) or only partially able (*ech2ibr10* mutant) to form ectopic metaxylem. This means that events upstream of the pericycle periclinal proliferation must be involved in the IBA priming action for ectopic metaxylem formation.

Under HF conditions, *shr* null mutant has been reported to be able to produce ectopic metaxylem in Arabidopsis dark-grown seedlings, and *scr* null mutant to do the same under IBA + Kin [1]. SHORT ROOT (SHR) and SCARECROW (SCR) are transcription factors of the GRAS family. SHR is essential for marking AR progenitor cells in Arabidopsis, and works forming a complex with SCR [1]. The complex produces the microRNA 165/166, which is involved in the destabilization of *HD-ZIP III* genes, which are determinant for metaxylem specification [7,13]. It has been hypothesized that in this way the SHR-SCR complex favours AR formation in the place of metaxylem formation [1]. In accordance with this hypothesis, the microRNA 165/166 is absent in *shr* and *scr* mutants [38], and HD-ZIP III mRNA is not degradated, but ectopic metaxylem is formed, in the miR165/166-resistant *phb-7d* mutant [2]. We hypothesize that IBA participates to the canalization of the pericycle cells toward either AR formation or metaxylem formation by modulating the gene expression that is related to the SHR-SCR complex activities. 

### 3.2. IAA Is Determinant for the Ectopic Protoxylem Formation

The role of IAA in promoting xylogenesis is widely known [14]. Present data clearly demonstrate that IAA induces the formation of ectopic protoxylem. Moreover, the response under IAA (±ACC) in the wild type and in the *ein3eil1* mutant, unable to transduce ethylene signal, demonstrate that the inductive action of exogenous IAA on the ectopic protoxylem does not need the cooperation of ethylene (Figure 2 and Figure 5). In addition, in all of the genotypes and treatments, except for rare cases in the IAA-treated *ech2ibr10* seedlings, the vascular connection of the ARPs with the hypocotyl vasculature was only constituted by protoxylem-like cells. Their formation is necessary for the ARP functioning, and, as for lateral root primordia, may be considered as a xylogenic event that must occur in the intervening cells of pericycle origin [39] to allow rapidly the conduction of water and mineral salts. The base of the forming ARP is a site of IAA accumulation [30,31], and the same occurs at the base of lateral roots, after IAA redistribution during primordium development [40]. Altogether, these results support the positive relationship between IAA and protoxylem-like formation. Genes of the protoxylem identity might be rapidly induced by IAA, irreversibly committing the pericycle derivative cells toward this program. This early canalization might be less expensive in terms of cell wall material production and assembly, and more rapid than metaxylem formation, for providing a xylary connection of the ARP to the parental vasculature. Moreover, it is known that IAA induces oxidative-stress during xylogenesis, thus enhancing the production of reactive oxygen species and nitrogen species in the target cells [18]. This stress might accelerate the cell death and cell wall thickening processes, which are both indispensable for xylary development [41], contributing also in this way to favour ectopic protoxylem vs. metaxylem.

### 3.3. Ethylene Is Required for the IBA-Induced Promotion of Ectopic Metaxylem

IBA is known to become active only in the target cell of its action [37]. Present data show that ectopic metaxylem is induced in *ech2ibr10* in the absence of exogenous hormones (Figure 6). This suggests a direct action of endogenous IBA on the pericycle-derived target cells, possibly through a process that is slower than the IAA-induced protoxylem formation, i.e., the process active in the wild type, where the IBA-to-IAA-conversion is functioning. The total absence of ectopic metaxylem in *ein3eil1* hypocotyls (Figure 5) clearly shows that endogenous/exogenous IBA requires ethylene to induce metaxylem-like element formation. 

Ethylene stimulates xylogenesis in *Zinnia elegans* cell cultures, positively affecting the rate of tracheary element differentiation by increasing the capacity of the cells to maintain the meristematic identity [25]. In accordance, ethylene increases the number of cambium cell layers in poplar trees [42], with a specific stimulatory role on cell division [24]. However, present results show that this is not the case for the basal hypocotyl of the Arabidopsis dark-grown seedlings, because, the ACC-derived ethylene did not cause a significant enhancement of proliferation in the wild type, and even highly reduced it in *ech2ibr10* mutant (Figure 3A).

All together, our data support that ethylene must be perceived by the EIN3EIL1 network for enhancing the IBA-induced ectopic metaxylem formation, but how IBA interacts with this network remains to be demonstrated. However, the increase in the number of the *ech2ibr10* seedlings with ectopic metaxylem formation under ACC alone treatment suggests that the IBA-interaction with the EIN3EIL1 network does not need the functioning of the conversion-into-IAA-machinery. 

### 3.4. IBA Is a Morphogen

How IBA functions independently of IAA is still unknown. Several lines of evidence, including the present results, hint at an action of IBA as a morphogen, at least for ectopic metaxylem formation, and for the realization of part of AR formation. It has been reported that a plant morphogen induces the acquisition of a new developmental fate in a cell or a group of cells by changes in its activity [43]. IAA has been considered as a morphogen in several contexts [44], however the concept seems true also for IBA, at least for the basal hypocotyl pericycle cells. In fact, the present research shows that IBA, as a morphogen, acts only in the derivatives of the pericycle cells of the basal hypocotyl in contact with the protoxylem of the vascular system. Its dual role changes the cellular developmental program, without inducing trans-differentiation, but after de-differentiation. Furthermore, we showed that IBA acts as an auxin per se in these cells for morphogenesis, and not as an IAA precursor.

## 4. Materials and Methods 

### 4.1. Plant Growth

Seeds of Col-0 ecotype of *Arabidopsis thaliana* (L.) Heynh and of its homozygous double mutants *ein3eil1* [45] and *ech2-1ibr10-1* [27] were sown, after sterilization, on square Petri plates (12 cm × 12 cm; 12 seeds per plate) containing full strength Murashige and Skoog (MS) [46] salts supplemented with 0.55 mM myo-inositol (Fluka, Buchs, Switzerland), 0.1 μM thiamine-HCl (Sigma-Aldrich, St. Louis, MO, USA), 1% (*w*/*v*) sucrose (Sigma-Aldrich) and 0.8% (*w*/*v*) agar (Sigma-Aldrich) (pH 5.7). As an alternative to this Hormone Free (HF) condition, either ACC (0.1 μM) (Sigma-Aldrich), or IBA (10 μM; Sigma-Aldrich), or IBA plus ACC (10 μM IBA plus 0.1 μM ACC) were added according to Veloccia et al. [16]. IAA, at 10 μM, with/without 0.1 μM ACC, was alternatively added.

Briefly, independently of the hormonal treatment, after sowing, the seeds were stratified for three days at 4 °C under continuous darkness and exposed to white light (intensity 100 μE·m^−2^·s^−1^) for about 6 h, to induce germination. Then, the plates were placed in vertical position under continuous darkness until 22 days after stratification (DAS), at 22 ± 2 °C, humidity 70%. 

### 4.2. Histological Analysis 

After 22 DAS, 30 seedlings per genotype and treatment were fixed, dehydrated, embedded in resin, longitudinally sectioned (8 μm thickness) with a Microm HM 350 SV microtome (Microm, Walldorf, Germany), and stained with 0.05% toluidine blue (all procedures according to Della Rovere et al. [30]). In all genotypes and treatments, the sections were preliminary carried out on portions of 5 mm in length excised all along the hypocotyl. Based on the absence of a xylogenic response in all of the portions except for the lowerest one, only the basal hypocotyl was examined in the further analyses, and the results here reported. Sections were observed with a Leica DMRB microscope, and images acquired with a DC500 camera (Leica, Wetzlar, Germany).

### 4.3. Statistical Analysis

Measures of the radial extension of the vascular system, including the de novo formed cells by pericycle periclinal proliferation, were carried out at the middle of the basal portion of the hypocotyl of each seedling, and were expressed as means (±SE). The number of ARs/ARPs present in the same basal portion of each seedling was also expressed as mean value (±SE). Two-way ANOVA (*p* < 0.05) was used to compare the effects of treatments and genotypes, and, if ANOVA showed significant effects, Tukey’s post-test was applied (GraphPad Prism 6.0, GraphPad Software, Inc., La Jolla, CA, USA). 

Experiments were repeated three times, with similar results (data of the second replicate are shown).

## Abbreviations

ACC	1-aminocyclopropane-1-carboxylic acid
AR	adventitious root
ARP	adventitious root primordium
DAS	days after stratification
HF	hormone free
IAA	indole-3-acetic acid
IBA	indole-3-butyric acid
